# Current Status of Gastric Cancer Screening and Future Perspectives

**DOI:** 10.1002/deo2.70148

**Published:** 2025-05-26

**Authors:** Chika Kusano, Fumiaki Ishibashi, Chikamasa Ichita, Takuji Gotoda

**Affiliations:** ^1^ Department of Medicine Division of Gastroenterology and Hepatology Kitasato University School of Medicine Sagamihara Japan; ^2^ Department of Gastroenterology International University of Health and Welfare Ichikawa Hospital Ichikawa Japan; ^3^ Gastroenterology Medicine Center Shonan Kamakura General Hospital Kamakura Japan; ^4^ Department of Gastroenterology Cancer Institute Hospital Tokyo Japan

**Keywords:** endoscopy, gastric cancer screening, *Helicobacter pylori* test, serum pepsinogen test, upper gastrointestinal contrast radiography

## Abstract

Gastric cancer (GC) remains a major global health concern, particularly in East Asia, Central Asia, and Eastern Europe, where its incidence and mortality rates are high. *Helicobacter pylori* infection is the primary cause of GC and leads to carcinogenic progression from nonatrophic gastritis to cancer. Although screening programs have been implemented in high‐risk countries, such as Japan and South Korea, comprehensive strategies remain limited globally. This study reviewed the status of GC screening worldwide and prevention strategies in regions with different risks. Various GC screening methods have been developed, including *H. pylori* serology, serum pepsinogen testing, upper gastrointestinal contrast radiography, and endoscopy. Endoscopic screening has shown superior sensitivity and specificity, reducing GC mortality by up to 47% in South Korea and demonstrating higher detection rates than upper gastrointestinal contrast radiography and pepsinogen testing. However, cost‐effectiveness remains a challenge, particularly in Western countries where the overall GC prevalence is lower. Risk stratification using a combination of *H. pylori* serology and pepsinogen testing has been adopted in Japan to optimize screening efficiency. Additionally, *H. pylori* eradication has been recognized as a cost‐effective strategy to reduce the incidence of GC with economic benefits demonstrated in Japan and other high‐risk regions. In the United States, targeted screening of high‐risk immigrant populations has been suggested to enhance cost‐effectiveness. GC screening strategies should consider developing epidemiological trends, cost‐effectiveness, and risk‐based approaches. Future efforts should focus on expanding targeted screening initiatives to high‐risk groups to improve early detection and survival rates.

## 1 Introduction

Gastric cancer (GC) is the sixth most common type of cancer and the third most common cause of cancer‐related deaths [1]. Most GC cases worldwide are thought to be caused by *Helicobacter pylori* infections and the resulting carcinogenic progression (Koehler's cascade) [2, 3, 4, 5]. In this model, chronic inflammation caused by the colonization of *H. pylori* leads to a series of precursor conditions that progress to nonatrophic gastritis, atrophic gastritis, intestinal metaplasia, dysplasia, and, finally, cancer. Other chronic inflammatory mechanisms (such as autoimmune gastritis) may also cause cancer progression.

Most cases are concentrated in East and Central Asia, Central and South America, and Eastern Europe [6, 7]. The average incidence rate in East Asia is 32.1 per 100,000 men and 13.2 per 100,000 women [6], whereas that in North America is 5.6 per 100,000 individuals. Half of all cases worldwide occur in China [8], reflecting the high incidence rate in the country (21 cases per 100,000 people) and its large population. Japan and South Korea are ranked second and third, respectively, in terms of incidence but 38th and 64th, respectively, in terms of mortality (Japan: 8 per 100,000 people; South Korea: 6 per 100,000 people) [9]. As will be discussed later, these two countries are unique in that they have organized nationwide GC screening programs. Globally, the 5‐year net survival rate for GC is generally <35% (including wealthy countries in North America and Western Europe), but it is >60% in Japan and South Korea [10].

Despite the high incidence and mortality rates of GC, the implementation of prevention programs by governments and healthcare systems is limited (mainly in East Asia). This study reviewed the current status of GC screening worldwide and prevention strategies in regions with different risks.

### 1.1 GC Screening Method

*H. pylori *serology, serum pepsinogen (PG) testing, upper gastrointestinal (UGI) contrast radiography, and endoscopy have been used for GC screening [11] An ideal screening method should be simple, safe, valid, and cost‐effective. No single method meets all these criteria; therefore, a combination of several methods is preferable for more effective detection of GC. The advantages and disadvantages of each screening method are summarized in (Table 1) [11, 12].

**TABLE 1:** Advantages and disadvantages of each screening method.

Method	Advantage	Disadvantage
Upper gastrointestinal contrast radiography	Moderate evidence	Exposure to radiation Requires endoscopic confirmation
Endoscopy	Most accurate Able to biopsy samples during performance	Invasive and expensive Requires trained professionals and equipment
*H. pylori* serology	Noninvasive	Very low sensitivity No detection of premalignant lesions
Serum pepsinogen testing	Noninvasive Acceptable sensitivity and specificity Predicts premalignant lesions	Optimal cutoff values affected by other factors (age, sex, race, *H. pylori* eradication) Low evidence Requires endoscopic confirmation

*Note*: H. pylori, *Helicobacter pylori*.

### 1.2 UGI Contrast Radiography

Three cohort studies (Table 2) and three case‐control studies (Table 3) reported a reduction in mortality from GC owing to radiographic screening [13, 14, 15, 16, 17, 18]. Case‐control studies have primarily evaluated the effectiveness of endoscopy [13, 17, 18]. These results are inconsistent with those of previous studies showing that radiographic examination has a significant impact.

**TABLE 2:** Results of cohort studies on radiographic screening.

Authors	K. J. Lee [14]	A. Miyamoto [15]	L. Rosero‐Bixby [16]
Publication year	2006	2006	2007
Country	Japan	Japan	Costa Rica
Number of the screening group	26 961	24 014	6206
Age of screening group (years)	49.2 ± 5.9 (mean)	Men 52.33 women 53.2 (mean)	64.3 (mean)
Follow‐up periods	13.1 years (average)	11 years	2–7 years
Relative risk, screened vs. not screened (95% CI)			
Gastric cancer incidence	1.06 (0.90–1.25)	0.94 (0.79–1.13)	‐
Gastric cancer mortality	0.52 (0.36–0.74)	0.54 (0.38–0.77)	0.42–0.52
All‐causes mortality	0.71 (0.65–0.78)	0.83 (0.77–0.90)	‐

Abbreviation: CI, confidence interval.

**TABLE 3:** Results of case−control studies on radiographic screening and endoscopic screening.

Author	C. Hamashima [13]	S. Matsumoto [17]	J. K. Jun [18]
Publication year	2013	2014	2017
Country	Japan	Japan	South Korea
Number of case subjects	410	13	44 095
Age of case subjects (years)	40–79 (range)	72 ± 10 (median ± standard deviation)	≥40
Number of control subjects	2292	130	176 380
Age of control subjects (years)	40–79 (range)	69 ± 10 (median ± standard deviation)	≥40
Odds ratio (95% CI)			
Radiographic screening	0.865 (0.631–1.185)		0.98 (0.95–1.01)
Endoscopic screening	0.695 (0.489–0.986)	0.206 (0.044–0.965)	0.53 (0.51–0.56)

Abbreviation: CI, confidence interval.

Cohort studies suggest a reduction in mortality of up to 40% owing to screening with radiographic examination [14, 15, 16]. However, in Japanese cohort studies, people with symptoms may have been included in the GC screening group, and the decision to undergo GC screening during the follow‐up period was left to the individual. Therefore, although good results have been obtained in Japanese studies, the reduction in mortality may have been overestimated.

The sensitivity and specificity of radiographic and endoscopic screening have been reported in Korea and Japan [19, 20]. A Korean study reported the sensitivity of radiographic screening to be 38.2% (95% confidence Interval [CI]: 35.9–40.5) for the first round of screening and 27.3% (95% CI: 22.6–32.0) for subsequent rounds [19]. A Japanese study reported the sensitivity of endoscopy to be 89.3% (95% CI: 71.8–97.7) for prevalence screening (first round) and 88.5% (95% CI: 66.4–97.2) for incidence screening (second round; Table 3) [20].

### 1.3 Endoscopic Screening

In 2017, a South Korean study provided evidence of a reduction in GC mortality due to endoscopic screening. A South Korean nested case‐control study based on a national database reported a 47% reduction in mortality from GC due to endoscopic screening [18]. In particular, the reduction in GC mortality owing to endoscopic screening was observed in the participants who underwent endoscopic screening within 1–4 years of the date of GC diagnosis and in the age group of 40–74 years.

A study conducted in Niigata and Tottori prefectures had a sufficient sample size to evaluate the reduction in mortality from GC due to endoscopic screening. The GC mortality was 30% lower in people who had undergone at least one endoscopic screening within 36 months (odds ratio 0.695, 95% CI: 0.489–0.986) [13]. However, patients who underwent radiographic screening showed no significant reduction in GC mortality.

Five studies calculated the test accuracy of endoscopic screening [19, 20, 21, 22, 23], but most of them did not have follow‐up data after obtaining negative results. A Korean study reported the sensitivity of endoscopic screening using this detection method to be 69.4% (95% CI: 66.4–72.4) for the first round of screening and 66.9% (95% CI: 59.8–74.0) for subsequent rounds [19]. A Japanese study reported the sensitivity of prevalence screening to be 95.5% (95% CI: 87.5–99.1) and the sensitivity of incidence screening to be 97.7 (95% CI: 91.9–99.7; Table 3) [20].

A systematic review and meta‐analysis that evaluated the impact of endoscopic screening on GC mortality and incidence [24] analyzed 10 studies from Asia (totaling 342,013 individuals) and demonstrated that endoscopic screening was associated with a 40% reduction in GC mortality (relative risk [RR], 0.60; 95% CI: 0.49–0.73). However, endoscopic screening and the incidence of GC showed no significant association (RR, 1.14; 95% CI: 0.93–1.40). A subgroup analysis showed significantly lower GC mortality compared with those associated with no screening (RR, 0.58; 95% CI: 0.48–0.70) and radiographic screening (RR, 0.33; 95% CI: 0.12–0.91). These findings suggest that endoscopic screening may reduce GC mortality.

Faria et al. conducted a systematic review and meta‐analysis showing that esophagogastroduodenoscopy (EGD) demonstrated a significantly higher detection rate for GC and early GC (EGC) in double‐arm studies compared to UGI series (UGIS), with odds ratios of 3.29 (95% CI: 2.49–4.35, I^2^ = 76%) and 5.50 (95% CI: 3.93–7.70, I^2^ = 0%), respectively [25]. In single‐arm studies, the pooled GC detection rate for EGD (0.55%, 95% CI: 0.39–0.75) was significantly higher than that for UGIS (0.19%, 95% CI: 0.10–0.31) and PG testing (0.10%, 95% CI: 0.05–0.16). Similarly, the EGC detection rate was also higher for EGD (0.48%, 95% CI: 0.34–0.65) than those for UGIS (0.08%, 95% CI: 0.04–0.13) and PG testing (0.10%, 95% CI: 0.04–0.19). Endoscopic screening may have higher detection rates of GC, and EGC than UGIS.

#### 1.3.1 *H. pylori* Serology

No studies have assessed the impact of the *H. pylori* antibody test on reducing GC mortality. *H. pylori* serology has limited use in GC screening because of its low sensitivity and failure to detect premalignant lesions [12]. Therefore, *H. pylori* serology is not an effective stand‐alone screening test for GC.

## 2 Serum PG Test

Several studies have investigated the effectiveness of PG testing for GC screening. Yanaoka et al. reported that, using a cut‐off value of PG I ≤70 and PG I/II ≤3.0, the sensitivity and specificity of the serum PG test was 58.7% (95% CI: 45.6–70.8) and 73.4% (95% CI: 72.1–74.6), respectively [26]. Meta‐analyses and cohort studies have demonstrated the utility of serum PG levels in assessing the risk of GC. A meta‐analysis of 300,000 patients showed that serum PG testing had a sensitivity and specificity of 77% and 73%, respectively, for GC screening using only the PGI/II ratio [27].

A case‐control study indicated that screening with serum PG testing was associated with a reduction in GC mortality, with odds ratios for death of 0.23 (95% CI: 0.06–0.92) and 0.37 (95% CI: 0.15–0.90) in individuals screened within 1 and 2 years, respectively [28]. However, despite these promising findings, evidence is insufficient to warrant the inclusion of serum PG testing in Japan's national GC screening program. Limitations of serum PG testing include variations in threshold values for PG I and the PG I/II ratio, as well as fluctuations in these markers based on factors such as age, sex, and ethnicity. Positive PG results often revert to negative after eradication therapy [29]. A previous study demonstrated that within 1–3 months after eradication, PG‐I and PG‐II levels in *H. pylori*‐positive patients decreased to levels comparable to those in *H. pylori*‐negative individuals, although the PG‐I/II ratio remained lower [30].

### 2.1 GC Risk Stratification

In Japan, a combined approach using *H. pylori* antibody and serum PG tests is widely employed and has been adopted as an alternative for GC screening. The likelihood of developing GC increases because of factors such as *H. pylori* infection and gastric mucosal atrophy [31]. Although this method has shown high sensitivity in predicting GC development, its specificity is low, leading to a high rate of false positives [32]. However, when paired with endoscopic screening, this could potentially allow for longer screening intervals in individuals at a low risk of GC.

One randomized controlled trial study investigated the GC detection rate during endoscopic screening based on serological risk stratification (ABC classification) [33, 34]. In this study, the cost‐effectiveness of annual GC screening using barium and endoscopic screening based on serological risk stratification (ABC classification) was compared. Overall, 1206 participants were randomly assigned and followed up for 5 years. In the Ba‐Endo group, annual barium screenings were conducted, whereas, in the ABC‐Endo group, endoscopic examinations were performed according to risk stratification. There was no significant difference in the GC detection rates between the two groups (full analysis, 1.0%; per‐protocol analysis, 3.4%). However, the proportion of GCs successfully treated with endoscopic resection alone was significantly higher in the ABC‐Endo group (full analysis, 90.9%) than in the Ba‐Endo group (full analysis, 41.6%), suggesting that endoscopic screening based on the ABC classification may enhance the effectiveness of endoscopic treatment for early stage GC. Group A may have included individuals with current or past *H. pylori* infection. According to reports, approximately 20% of the individuals in Group A had a current or past *H. pylori* infection. *H. pylori* antibodies often showed intermediate values (3–9.9 U/mL). The optimal cutoff value for PG I in determining past *H. pylori* infection was ≤31.2 ng/mL, while that for the PG I/II ratio was ≤4.6 [35]. As mentioned above, the ABC classification has limitations in assessing GC risk. However, if the *H. pylori* infection rate decreases and most screening participants are *H. pylori*‐negative, it may be possible to assess risk based solely on the presence or absence of *H. pylori* infection (current or past).

### 2.2 Cost‐Effectiveness of *H. pylori* Testing and Eradication

Multiple simulation studies have assessed the cost‐effectiveness of *H. pylori* test‐and‐treat strategies for GC prevention in various Western countries, including the United States (US) [36, 37], UK [38, 39], Canada [40], New Zealand [41], and Finland [42]. These studies typically targeted men aged ≥50 years, with the *H. pylori* prevalence ranging from 30 to 40% [43]. All studies evaluated the effectiveness of a one‐time *H. pylori *serological test, with some comparing it against other testing methods, such as fecal antigen testing and C‐urea breath tests [40]. Despite the differences in model assumptions, studies have consistently concluded that the test‐and‐treat approach is cost‐effective in reducing GC mortality. The cost per quality‐adjusted life years (QALY) gained was generally under $25,000, and certain populations, such as men, Japanese Americans, and African Americans, showed even greater cost‐effectiveness [44].

A Japanese study that evaluated *H. pylori* eradication as a national strategy for GC prevention also demonstrated substantial health and economic benefits [45]. Using a cohort model, this study showed that *H. pylori* eradication was more effective and cost‐effective than no eradication across all age groups. From 2013 to 2019, the strategy saved $3.75 billion, increased life‐years and QALYs, and prevented a significant number of GC cases and deaths. For a larger cohort, the strategy could potentially save over $14 billion while preventing over a million cases of GC [46] (Table 4). These findings strongly suggest the adoption of a nationwide *H. pylori *eradication strategy in countries with a high GC incidence.

**TABLE 4:** The cost‐effectiveness of *H. pylori* testing/eradication and endoscopic screening.

Strategy (reference number)	Cost‐effectiveness (°/×)	Summary
*H. pylori* test‐and‐treat (Western countries) [36, 37, 38, 39, 40, 41, 42, 43, 44]	°	Consistently cost‐effective across the US, UK, Canada, New Zealand, and Finland; The cost per QALY is generally under $25,000. The greater benefit was observed among men, Japanese Americans, and African Americans.
*H. pylori* eradication (Japan) [45, 46]	°	Substantial health and economic benefits. $3.75 billion saved (2013–2019); potential savings exceeding $14 billion Prevention of over one million gastric cancer cases.
Endoscopic screening (general US population) [47, 48]	×	Not cost‐effective; One‐time screening at age 50 costs $115,664 per QALY, exceeding the $100,000 threshold.
Endoscopic screening (Asian Americans) [48, 49]	°	Cost‐effective when stratified by race/ethnicity; $71,451 per QALY for Asian Americans, with the most favorable ICERs among Chinese, Japanese, and Korean Americans.
Endoscopic Screening (Low *H. pylori* Prevalence Population, Japan) [50]	°	Most cost‐effective strategy: screening every four years starting at age 40, with the highest net monetary benefit (97,401,578 yen).

Abbreviations: *H. pylori*, *Helicobacter pylori*; QALY, quality‐adjusted life years.

### 2.3 Cost‐Effectiveness of Endoscopic Screening

Endoscopic GC screening is not cost‐effective in the general US population. One‐time screening at the age of 50 was found to cost $115,664 per QALY, which exceeds the $100,000 threshold for cost‐effectiveness (Table 4) [47]. These studies did not consider race, ethnicity, or the presence of gastric intestinal metaplasia, which could influence cost‐effectiveness [48]. However, when costs were stratified by race and ethnicity, upper endoscopic screening was more cost‐effective in certain groups. Asian Americans, Hispanics, and non‐Hispanic blacks benefited from a single upper endoscopy at the age of 50 years, with varying levels of cost‐effectiveness depending on ethnicity. For Asian Americans, the cost‐effectiveness was $71,451/QALY, with Chinese, Japanese, and Korean Americans having the lowest incremental cost‐effectiveness ratios (ICERs) [48, 49] (Table 4).

Ishibashi et al. evaluated the optimal endoscopic screening strategy for GC in a population with a low prevalence of *H. pylori* infection [50]. The participants had a mean age of 54.5 years, with 74.4% of *H. pylori*‐naïve participants and 94.2% of participants testing negative for intestinal metaplasia. A Markov model was constructed to compare the cost‐effectiveness of the 15 screening strategies with different starting ages (40/50/60 years) and screening intervals (1/2/3/4/5 years). The results showed that endoscopic screening every 4 years, starting at the age of 40 years, had the highest net monetary benefit (97,401,578 yen; Table 4). This strategy consistently demonstrated superior ICER in both Monte Carlo simulations and sensitivity analyses.

### 2.4 Conclusion and Future Perspective

The future of GC screening in Japan is expected to follow a downward trend in the incidence of GC because the younger generation has lower rates of *H. pylori *infection. According to Japanese guidelines, population‐based GC screening is recommended by balancing the benefits and harms, with criteria based on the number needed to screen and that needed to recall. When the RR for mortality reduction is set at 0.6, screening is recommended for men aged ≥55 years and women aged ≥65 years. As the RR increases to 0.7 or 0.8, screening is recommended for men aged ≥65 years and women aged ≥75 years. Therefore, GC screening will likely not be recommended for men and women in their 40s and early 50s, even by 2035 [51].

In contrast, in the US, comprehensive GC screening is recommended for high‐risk groups, particularly immigrants from countries with high incidence rates of GC. Screening should focus on individuals with risk factors such as *H. pylori *infection, family history, and gastric atrophy/IM [52]. Policymakers should also consider offering these screenings to high‐risk populations, even in the absence of symptoms. Many Western countries that accept large numbers of immigrants from high‐incidence countries could benefit from such studies. In Japan, there is a need to consider new approaches for the screening of high‐risk groups.

## Conflicts of Interest

This study was supported by grants (Grant Number 24EA1001) from the Ministry of Health, Labor and Welfare of Japan. Chika Kusano, Fumiaki Ishibashi, and Takuji Gotoda received a speaker honorarium from Olympus Marketing Inc. Fumiaki Ishibashi and Takuji Gotoda received a speaker honorarium from FUJIFILM Medical.Co, too.
